# Molecular Markers for Prostate Cancer in Formalin-Fixed Paraffin-Embedded Tissues

**DOI:** 10.1155/2013/283635

**Published:** 2013-11-25

**Authors:** Tamara Sequeiros, Marta García, Melania Montes, Mireia Oliván, Marina Rigau, Eva Colás, Inés de Torres, Juan Morote, Jaume Reventós, Andreas Doll

**Affiliations:** Research Unit in Biomedicine and Translational Oncology, Research Institute Vall Hebron University Hospital (VHIR) and Universitat Autònoma de Barcelona (UAB), 08035 Barcelona, Spain

## Abstract

Prostate cancer (PCa) is the most frequently diagnosed type of cancer in developed countries. The decisive method of diagnosis is based on the results of biopsies, morphologically evaluated to determine the presence or absence of cancer. Although this approach leads to a confident diagnosis in most cases, it can be improved by using the molecular markers present in the tissue. Both miRNAs and proteins are considered excellent candidates for biomarkers in formalin-fixed paraffin-embedded (FFPE) tissues, due to their stability over long periods of time. In the last few years, a concerted effort has been made to develop the necessary tools for their reliable measurement in these types of samples. Furthermore, the use of these kinds of markers may also help in establishing tumor grade and aggressiveness, as well as predicting the possible outcomes in each particular case for the different treatments available. This would aid clinicians in the decision-making process. In this review, we attempt to summarize and discuss the potential use of microRNA and protein profiles in FFPE tissue samples as markers to better predict PCa diagnosis, progression, and response to therapy.

## 1. Introduction

Prostate cancer (PCa) is the most commonly diagnosed cancer and the second leading cause of cancer death among European and American men [1]. The current screening method to diagnose PCa is based on a measurement of serum prostate specific antigen (PSA) levels and a digital rectal examination (DRE), while a decisive diagnosis is based on the results of transrectal, ultrasound-guided prostate biopsies (PBs). 

The introduction of the serum PSA test in the late 1980s has led to an increase in the detection of new PCa cases [2]. However, serum PSA has some well-recognized limitations; it lacks diagnostic specificity and prognostic value, and it leads to a high rate of false positives [3]. This lack of specificity is associated with an increased percentage of negative PB and the overdiagnosis of many indolent tumors, resulting in the overtreatment of patients. Consequently, there exists urgent need to find more effective and specific PCa detection markers. 

The management of diagnosed PCa is crucially dependent on the presentation of the disease [4]. By nature, PCa progresses slowly and can be treated effectively with early detection by radical prostatectomy (RP); however, patients diagnosed with high risk PCa or metastatic disease have a 50% risk of disease progression 5 years after surgery [5]. For this reason, these patients are provided with closer follow-up and more intensive treatment by adjuvant therapy to avoid local and distant disease, usually radiation therapy (RT) and androgen deprivation therapy (ADT) [6]. To help clinicians choose the best treatment approach for each particular case, it is critical to identify markers that distinguish indolent cases of PCa from those that will progress and metastasize. 

In the past few years, thanks to current advancements in proteomics, RNA and DNA microarrays, immunohistochemical (IHC) staining, and other biotechnologies, new approaches have been applied to identify and validate more accurate diagnostic and prognostic biomarkers in tissue samples [7]. 

## 2. Biomarker Discovery in FFPE Tissue

Although fresh-frozen (FF) tissue remains the gold standard for extraction and large-scale profiling in genomic and proteomic studies, analyzing molecules in formalin-fixed paraffin-embedded (FFPE) tissues is gaining increased interest. This interest is primarily driven by the fact that the process of creating FFPE tissue is the most common technique used by clinical and/or research pathologists for tissue processing, evaluation, diagnostics, immunoanalysis, preservation, and archiving. These archived FFPE tissues may provide wealth of information when used in retrospective molecular studies that focus on molecular profiling and biomarker research [8]. It is true that the quantity and quality of proteins, nucleic acids, and metabolites obtained from FFPE tissues are inferior to the extraction efficiency obtained from FF tissues [8, 9]. However, the use of FFPE samples in molecular expression analysis studies presents some great advantages. For example, these types of samples are available and readily accessible in vast quantities. The cost associated with their storage is low, as well, and the significant association between pathological and clinical annotations makes FFPE tissue an attractive specimen for biomarker discovery. Nevertheless, when working with nucleic acids, FFPE samples present some drawbacks as RNA and DNA can be degraded and modified by the fixation process and, independently, degraded with time. 

In the late 1990s, tissue microarray (TMA) technology revolutionized the investigation of potential prognostic and predictive biomarkers [10, 11]. TMAs are commonly used to study tissue morphology, the expression of proteins or genes, and chromosomal aberrations using IHC and *in situ* hybridization (ISH) [11]. The combination of TMAs and clinically annotated samples is useful when studying panels of biomarkers under identical experimental conditions, as well as in the development of prognostic or predictive models for patient outcome [12]. Despite these considerable advantages, the conventional construction of TMAs is often meticulous, laborious, and time consuming. Recently, Zlobec et al. [11] proposed the term, *next-generation tissue microarrays* (ngTMAs), to define a combination of cutting-edge digital pathology, automated TMAs, and histopathological expertise, which would ultimately benefit the further optimization of biomarker research.

The purpose of this review is to examine the potential use of previously described, small noncoding RNAs, microRNAs, and protein profiles as markers that may help predict PCa diagnosis, progression, and response to therapy in prostate FFPE specimens. To date, only a few of these markers have achieved widespread clinical use. 

### 2.1. MicroRNAs as Biomarkers in Prostate FFPE Samples

In recent years, evidence has accumulated showing that small noncoding RNAs are used in a conserved manner to regulate key developmental events. At least four classes of regulatory, small noncoding RNAs have been described, including microRNAs (miRNAs), short interfering RNAs (siRNA), repeat-associated small interfering RNAs (rasiRNAs), and piwi-interacting RNAs (piRNAs) [13]. Among these small RNAs, the miRNAs, which are the most phylogenetically conserved, posttranscriptionally regulate the genes involved in several physiological and pathological processes [14–16]. Therefore, their aberrant expression results in a variety of pathological events, such as cancer [17, 18]. Generally, the importance of miRNAs in cancer is emphasized by the fact that around 50% of all miRNA genes are positioned in so-called fragile sites, genomic regions that are associated with repeated changes that occur in cancer [18, 19]. Moreover, miRNAs are attractive candidates as multifunctional regulators of disease progression, because one miRNA can regulate an entire set of genes [20]. It is believed that miRNAs regulate about 30% of all protein-coding human genes [21–23]. Finally, several miRNAs and their targets have been found to be aberrantly expressed in PCa [18, 24–26]. For that reason, certain miRNAs are now considered valuable biomarkers for diagnosis, prognosis, and the classification of PCa [27, 28]. 

Furthermore, miRNAs present several advantageous features that make them a source of potential cancer biomarkers in FFPE tissues. First, unlike other nucleic acids, miRNAs are potentially more robust. Due to their small size and protection by the RISC complex, which makes them resistant to endogenous RNase activity, they are less affected by FFPE-dependent degradation [29–32]. Second, their expression levels can be measured reliably in FFPE tissue samples, and only minute quantities of RNA are needed to establish their expression using reliable, quantitative PCR amplification strategies [33]. Third, miRNA expression profiles are not dependent on the preservation of the specimen's architecture and cellular arrangement or the degree of cellular degeneration [29]. Finally, a good correlation between the expression profiles of FF and FFPE samples with miRNAs and messenger RNAs (mRNAs) has been observed and reported in liver [34], glioblastoma [35], and PCa specimens [36]. Therefore, the expression profiling of miRNAs is an accurate and robust method for the molecular analysis of archived clinical specimens, which potentially extends the use of miRNAs as new diagnostic, prognostic, and treatment response biomarkers [30]. Currently, high-throughput screening methods, such as microarrays, can be applied to detect miRNAs in prostate FFPE tissues; however, quantitative real-time reverse-transcription PCR (qRT-PCR) is still one of the most common high sensitivity and specificity methods used to detect low miRNA levels [37].

In recent years, specific miRNA signatures for PCa have been described in several studies [27, 28, 38–44], suggesting that miRNAs or miRNA profiles can be used as diagnostic and prognostic markers for this disease [45]. These markers exhibit distinct abilities to detect PCa and to predict disease course. Some of them are listed in Table 1 and discussed below.

The downregulation of miRNAs is the most frequently observed phenomenon in cancer, suggesting that they function as tumor suppressor genes [46]. Loss of mir-145 expression has been reported in many human cancers [47, 48], including PCa [49]. The downregulation of miR-145 has been associated with an aggressive phenotype and poor prognosis in PCa [50]. Peng et al. [51] reported that in FFPE specimens the downregulation of miR-143 and miR-145 was negatively correlated to bone metastasis, Gleason score, and the level of free PSA in primary PCa. Recently, Suh et al. [49] documented that the downregulation of miR-145 in PCa could play a role in cancer initiation, and that the mechanism for its regulation is mediated by DNA methylation and p53 mutation pathways (a protein marker discussed later in this review).

Some of the most frequently mentioned miRNAs that appear downregulated in PCa are members of the let-7 family [45]. This family appears to play a key role in the recurrence and progression of PCa by maintaining and regulating the molecular features of cancer stem cells (CSCs) or cancer stem-like cells [52]. Loss of the let-7 family has been found in human PCa FFPE tissue specimens, especially in higher Gleason grade tumors, and it has been strongly linked to the acquisition of epithelial to mesenchymal transition (EMT) phenotype [52] and to the negative regulation of RAS protein [53]. These data suggest that inducing the re-expression of miRNAs from the let-7 family could represent a new therapeutic approach for aggressive PCa. A very recent report [45] identified let-7b as an independent prognostic marker for biochemical recurrence (BCR) and clinical failure in high-risk PCa. Furthermore, alterations in the expression profile of miR-let7c, miR-100, and miR-218 have been described during the progression from localized to metastatic PCa [46, 54].

MiR-221 is also a strongly downregulated miRNA in PCa, as clearly confirmed by several miRNA expression studies of PCa [28, 39, 40, 55]. Spahn et al. [55] identified a miRNA profile, (including miR-221, which has been related to metastasis) comparing the miRNA expression patterns in primary carcinoma and lymph node metastatic tissue. Moreover, the downregulation of miR-221 was associated with clinicopathological parameters, including Gleason score and clinical recurrence. These results suggest that miR-221 could be a novel prognostic indicator in high risk PCa, and it could be clinically useful for advising additional therapeutic strategies to PCa patients.

MiR-200a, a member of the miR-200 family, has been implicated in the regulation of EMT and postulated to be hijacked by cancer cells during tumor metastasis [56]. Barron et al. [56] showed downregulation of miR-200a in PCa tissue from men who relapsed compared to those who did not. Moreover, they also observed reduced expression of the other two miRNAs in the miR-200a cluster (miR-200b and miR-429), suggesting that those miRNAs are jointly repressed in patients suffering from relapsing disease. Although miR-200a did not demonstrate adequate sensitivity or specificity to serve as a reliable relapse predictor, surely it could be considered a valuable addition to a panel of biomarkers. These findings are supported by other recent studies, which link miR-200 family members to PCa progression [52]. 

On the other hand, overexpressed miRNAs in cancer have occasionally been observed [38, 57]. These may function as oncogenes and promote cancer development by negatively regulating tumor suppressor genes and/or genes that control cell differentiation or apoptosis. 

The overexpression of miR-183, miR-96 and miR-182, individually or as a cluster, has been reported in cancer [58], including PCa [59, 60]. In particular, mir-182-5p has been described as an oncogene in several cancers [61–64]. Its expression was found to be significantly higher in PCa tissues compared to normal prostate tissues [59, 60, 65], and it has been associated with shorter overall survival in PCa patients. Recently, Tsuchiyama et al. [66] demonstrated that in FFPE tissue samples, the expressions of miR-182-5p, as well as mir-31-5p and mir-205-5p, were significantly higher in high grade tumors compared to those of intermediate grade. This would suggest that miR-182-5p is a useful marker for high grade PCa.

Long et al. [67], analyzing the expression profiles of 70 archived FFPE tumor specimens, showed that miR-519d and miR-647 could serve as biomarkers to discriminate between patients with and without BCR following RP. MiR-519d was positively associated with the risk of BCR, while miR-647 presented a negative association.

### 2.2. Protein Biomarkers in Prostate FFPE Samples

The diagnosis of PCa in histopathological specimens is based mainly on a combination of architectural and cellular atypia, and in a vast majority of cases, a confident diagnosis can be made based on morphology alone. However, in some cases, the morphological findings are insufficient for a conclusive diagnosis, either because the atypia is too mild or the atypical focus is too small. The diagnostic accuracy of a morphology-based diagnosis can be improved in some cases by IHC that uses one or several biomarkers, either on consecutive sections or by the use of stained marker cocktails [68]. 

The detection of protein biomarkers by IHC presents multiple advantages, due to their high stability in FFPE tissues compared to other molecules. Multiple reports have shown that proteins and even protein modifications, such as phosphorylations, are maintained and can be determined years later by IHC [69]. In the last two decades, the ability to detect antigens in tissue sections has improved dramatically, mainly by countering the deleterious effects of formaldehyde with antigen retrieval and increasing the sensitivity of the detection systems [70]. From a clinical perspective, the IHC method correlates molecular detail to histopathological changes found in patient-derived tissues. Consequently, IHC is an excellent, simple, and effective technique for the detection of molecular biomarkers in FFPE samples. Currently, the IHC method is considered one of the pillars of modern diagnostic pathology and a fundamental research tool in both pathology and translational research laboratories [71]. Some of the protein markers used for PCa diagnosis, prognosis, and response to therapy are listed in Table 2.

It is well known that despite the fact that it is neither organ nor cancer specific, PSA is the most important, accurate, and clinically useful biochemical marker in the prostate [72–74]. As proved using IHC techniques, PSA expression is localized to the differentiated, secretory columnar cells of the glandular epithelium [75]. The apical portion of the epithelial cell cytoplasm shows more intense staining than the lower part, whereas the basal cells, transitional epithelium, or stromal cells do not express PSA. The PSA protein is strongly expressed, both in normal and neoplastic prostatic tissue; however, as evidenced by IHC staining, it is expressed less in cancer than in benign epithelium, and its expression decreases with the decreasing differentiation of PCa [76, 77]. Although IHC detection of PSA is still widely used to identify metastatic prostatic adenocarcinoma, PSA may not be expressed in some poorly differentiated prostatic carcinomas [78–80]. Its immunoreactivity has also been found in some nonprostatic tissues [81–83], making it useless, in such cases, for confirming prostatic origin. 

Recently, novel marker proteins that are preferentially expressed in prostate tissue have been identified. Prostein (P501S), a prostate-specific marker that is expressed in the cytoplasm of benign and malignant prostatic glandular cells, is one of them. Owing to its high specificity and the fact that a large majority of metastatic prostatic adenocarcinomas are prostein positive (99%), the combined use of P501S with PSA could be a good marker for demonstrating prostatic origin in metastatic PCa [84, 85].

Several studies using FFPE samples have shown that prostate stem cell antigen (PSCA), a prostate-specific gene, is expressed in most PCa specimens. Its levels are positively correlated with Gleason grade, tumor stage, progression to androgen-independence, and BCR and have also been found to be particularly elevated in bone metastasis [86–88]. All these data suggest that PSCA may be useful in prognosis and may be a promising molecular target in the diagnosis and treatment of patients with metastatic PCa. Another marker shown to be specifically increased in PCa epithelia, compared to benign epithelia, is *α*-methylacyl-CoA racemase (AMACR or P504S) [89–91]. P504S is a PCa-specific gene that encodes a protein involved in the beta-oxidation of branched chain fatty acids. It is a sensitive and specific marker for prostatic carcinoma in FFPE tissues [92, 93]. Both high grade and low-grade PCa show strong cytoplasmic staining; however, a significant decrease in AMACR protein expression has been observed in cases of metastatic hormone-refractory disease compared with clinically localized PCa samples [94].

A useful IHC biomarker for PCa may be cysteine-rich secretory protein 3 (CRISP-3), also known as the specific granule protein of 28 kDa (SGP28). CRISP-3 was found to be associated with high Gleason grade with elevated intensity, and it was also overexpressed in high-grade prostatic intraepithelial neoplasia (HG-PIN), which indicates that CRISP-3 could be a marker for early cancer development [95, 96]. In addition, patients positive for CRISP-3 have smaller recurrence-free probabilities after RP [97]. Another protein-based candidate for PCa diagnosis is Golgi membrane protein 1 (GOLM1, also known as GP73 and GOLPH2), a transmembrane protein expressed in the epithelial cells of many human tissues [98]. Several studies have recently reported the upregulation of this protein in malignant prostate tissue, suggesting GOLM1 as an additional ancillary positive marker for the tissue-based diagnosis of PCa [99, 100]. On the other hand, PCa tissues, even those of high grade, only rarely express high-molecular-weight cytokeratin (HMWCK, sometimes also referred to as 34*β*E12, an anti-cytokeratin antibody, clone-specific for high-molecular-weight cytokeratins (cytokeratins 1, 5, 10, and 14)). That makes this marker a useful adjunct in the diagnosis of PCa [101, 102]. HMWCK immunoreactivity in benign glands is localized to the cytoplasm of basal cells and is negative in PCa. It is a particularly sensitive marker for the differentiation of PCa from high grade invasive urothelial carcinoma, where it can be detected by IHC [103–105]. Even though HMWCK labeling of PCa cells is uncommon, p63 has been reported to be even more specific than HMWCK as a marker for the basal cell nuclei of benign glands and with less tumor cell labeling [106]. 

The use of cocktails that include different antibodies as a routine test overcomes the problems of studying small lesions in prostate needle biopsies with multiple IHC stains [107, 108]. For this reason, many studies have focused on attempting new combinations of markers that could improve the diagnostic process. For example, the overexpression of AMACR, in combination with the absence of basal cell markers, such as HMWCK or p63, is typical of classic acinar prostatic adenocarcinoma. Its detection has been shown to be of great value in combatting morphologically suspicious cases, as well as significantly increasing diagnostic accuracy in PCa [91, 109–112]. Furthermore, several studies have compared the usefulness of a three-marker cocktail of antibodies for detecting PCa. These cocktails incorporated AMACR (positive in malignant glands), p63 (nuclear staining in basal cells of nonmalignant glands), and HMWCK (cytoplasmic staining in basal cells of nonmalignant glands), along with the traditional two-marker cocktail. These studies all concluded that adding an extra basal cell marker to the traditional two-antibody cocktail significantly improved specificity for the detection of PCa in limited needle biopsy material [107, 113, 114].

Phosphatase and tensin homolog on chromosome 10 (PTEN) has been described as one of the most frequently lost tumor suppressor genes in human cancers. Its expression is lost in more than two thirds of patients with advanced/aggressive PCa. Moreover, genomic and proteomic PTEN loss has been associated with tumor progression and poor prognosis in PCa [115–117]. In patients with clinically localized PCa, who were treated by RP, decreased PTEN expression has also been associated with an increased risk of recurrence and decreased time to metastasis [115, 118]. Choucair et al. [119] examined the deletion status of PTEN and the androgen receptor (AR) expression levels in FFPE PCa samples and found that PTEN genomic deletion predicts PCa recurrence. It was also associated with low AR expression and transcriptional activity.

Expression of the human Ki67 protein is known to be strictly associated with cell proliferation. The fraction of Ki67-positive tumor cells (the Ki67 labeling index, Ki67 LI) is often in correlation with the clinical course of the disease, including cases of PCa, where cell proliferation evaluated by Ki-67 increases from localized PCa to metastasis [54, 120]. A high Ki67 LI is independently associated with seminal vesicle infiltration and postoperative Gleason score. More importantly, the Ki67 LI can predict biochemical recurrence, particularly in the subgroup of patients with only a small amount of tumor in the biopsy. The Ki67 LI could also be a valid predictor of recurrence-free survival after radical prostatectomy [121, 122]. 

Cyclooxygenase-2 (COX-2) is also highly expressed in a number of human cancers and cancer cell lines, including PCa [123]. The potential roles of COX-2 in tumor related processes, such as tumorigenesis, angiogenesis [123–125], and radiation treatment resistance, make this an attractive biomarker candidate and potential therapeutic target [126–129]. Increased COX-2 expression is associated with biochemical failure and distant metastasis; therefore, it could be useful in identifying patients who require more aggressive therapy [130].

Recently discovered members of the B7-CD28 family, B7-H3, and B7x, were evaluated by IHC on pathological specimens from clinically localized PCa patients treated by RP [131, 132]. These studies concluded that B7-H3 was uniformly and aberrantly expressed in PCa and correlated to the worst clinical outcomes of this disease [131]. Therefore, B7-H3 could represent an independent predictor of cancer progression following surgery. Moreover, B7-H3 may encompass a novel diagnostic and potentially therapeutic target for the clinical management of PCa [131]. Another IHC study performed on tissue microarray sections using anti-B7-H3 and anti-B7x corroborated that these proteins were abundantly expressed in PCa and were associated with the spread of disease and poor outcome [133].

It has been suggested that p53 accumulation or *TP53* mutation could be used as both prognostic and predictive biomarkers. This is the most commonly mutated gene in human cancer and has been associated with poor prognosis in multiple and distinct types of cancer, including PCa [124, 134]. Abnormal p53 expression is a significant prognostic factor for patients with PCa, who have undergone short-term ADT and/or RT. It has also been suggested that long-term ADT may significantly improve the cause-specific survival for those with abnormal p53 [135]. Another biomarker associated with higher Gleason scores and lower biochemical-free survival in patients with advanced PCa undergoing ADT or RT is the gene product of the apoptosis regulator Bcl-2. The relative amounts of Bcl-2 and/or BAX, a proapoptotic protein from the same family, have been shown to correlate with tumor aggressiveness and radiation resistance in PCa [124, 136–138]. These data suggest that Bcl-2 expression could be used to inform the choice of ADT or RT dosage in individual patients [139–141].

The RI-alpha regulatory subunit of protein kinase A type 1 (PKA) is associated with active cell growth and neoplastic transformation. Its overexpression has been found to be predictive of outcome in PCa patients treated with RT and short-term ADT and is considered a potentially useful biomarker for identifying high risk PCa patients [142]. Another study from the same group showed that its overexpression was associated with an increased risk of failure after ADT and RT, suggesting that novel strategies may be needed for patients with tumors presenting high PKA levels [124, 143].

Most of the conventional prostate adenocarcinomas display focal neuroendocrine (NE) differentiation at diagnosis. The NE phenotype is emerging as an important factor in the evolution of PCa, since it seems to be implicated in the development of resistance to ADT [144]. Chromogranin A (CgA) appears to be the most sensitive marker, and it is the most frequently used marker for detecting the NE phenotype by IHC [145]. It has a strong association with pathological tumor stage, since its expression levels are higher in poorly differentiated carcinomas. It has also been identified as an additional prognostic marker after RP [146, 147].

## 3. Conclusions

For a long time, PSA screening for PCa has been controversial. Although the PSA test is simple and safe and has an acceptable sensitivity and specificity, the implementation of PSA screening for PCa costs nearly double is associated with a high risk of overdiagnosis, and as a consequence presents the inevitable side effects that arise from unnecessary treatment [148]. The current method of definitive diagnosis for PCa typically relies on the morphological findings in the biopsied tissue, which, even for an experienced pathologist, can be difficult to classify. This method could be improved by using one or several molecular markers, which would help the pathologist to make the final diagnostic decision and differentiate between clinically significant and clinically insignificant disease. Thus, novel markers are required to improve the specificity of PCa diagnosis and to fully characterize the heterogeneity of prostate tumor phenotypes. These markers will more accurately assess the diagnosis and prognosis of patients with PCa [149]. This strategy would play an important role in achieving better patient management in the clinical practice. It would also assure a decrease in the number of repeat biopsies and faster identification of those patients who require more aggressive therapy. It is evident that there exists urgent need to identify better biomarkers for PCa presence, progression, and response to treatment, in order to avoid unnecessary overtreatment and accurately predict disease outcome.

Recently, several attempts have been made to identify novel biomarkers for PCa, though the results were largely inconclusive [21]. However, several studies mentioned in this review have demonstrated that it is possible to find differentially expressed miRNAs and proteins that can distinguish between normal and malignant prostate tissues and can be used to classify and correctly diagnose even poorly differentiated tumor samples. Thus, both miRNA and protein expression profiles have potential as tools for the diagnosis and prognosis of cancer.

New technologies will be of great assistance in the application of these promising biomarkers to routine practice. An important challenge in cancer diagnostics will be to assay multiple parameters in a single slide when tissue quantities are limited. The development of multiplexed assays that maximize the yield of information from a small biopsy will help meet a critical challenge to current biomarker research. Accordingly, the implementation of ISH, in combination with IHC for the detection of clinically important miRNAs and protein markers in fixed specimens, now provides a fluorescence-based multicolor ISH/IHC assay. This assay is rapid, sensitive, and compatible with current automated clinical IHC assays and provides spatial characterization of miRNA expression [150, 151]. Thanks to this combined ISH/IHC assay, miRNA and protein biomarkers can now be used together in detecting, classifying, diagnosing, prognosing, and treating cancer. For multiplexing protein panels Matrix-assisted laser desorption ionization (MALDI) imaging mass spectrometry (IMS) appears as a powerful tool for the generation of multidimensional spatial expression maps of biomolecules directly from a FFPE tissue section. From a clinical proteomics perspective, this method correlates molecular detail to histopathological changes found in patient-derived tissues. Targeted IMS, by the incorporation of laser-reactive molecular tags onto antibodies, aptamers, and other affinity molecules, enables analysis of specific molecules or a class of molecules. The integration of MALDI-IMS methods into existing clinical pathology laboratory practices could prove beneficial to diagnostics [152]. 

New strategies for the detection of PCa in biopsies material are also being developed. For example, a recent study described a new type of bioactive membrane vesicle (with the proposed name “large oncosomes”) which can originate from tumor cells and is related to high Gleason grade and metastatic disease. They suggest that the detection of these pathological features could be adapted for routine histopathological analysis [153].

In this review, we have attempted to provide examples of potential miRNA and protein markers that have been discovered in prostate FFPE tissues, which may be of clinical benefit in PCa detection, prognosis, and/or prediction. Clearly, it would be beneficial to concentrate future efforts on the discovery and further study of the molecular mechanisms and regulatory pathways associated with PCa. This will help to improve the design and target selection of new therapeutic strategies. In summary, the development of novel and clinically relevant biomarkers in FFPE tissues for PCa diagnosis, prognosis, and prediction could contribute to the optimal identification and treatment of this disease.

## Figures and Tables

**Table 1:** miRNAs as PCa biomarkers in FFPE tissue.

miRNA	Clinical significance	References
Let-7 family	Diagnosis, prognosis (↓)	[45, 52]
miR-17	Diagnosis, prognosis (↓)	[154, 155]
miR-19a	Diagnosis	[156]
miR-20a/b	Diagnosis	[154]
miR-21	Prognosis (↑), treatment outcome	[157]
miR-25	Diagnosis	[156]
miR-26a	Diagnosis (↓)	[21]
miR-29a	Diagnosis (↓)	[21, 158]
miR-29b	Diagnosis (↓)	[159]
miR-31-5p	Diagnosis (↓)	[66]
miR-30d	Diagnosis (↑)	[21]
miR-34a	Diagnosis (↓)	[21, 160]
miR-34c-5p	Diagnosis (↓)	[66]
miR-93	Diagnosis	[154]
miR-101	Diagnosis	[154]
miR-106a	Diagnosis	[154]
miR-125b	Diagnosis (↑)	[25]
miR-126	Diagnosis (↓)	[21]
miR-132	Prognosis (↓), treatment outcome	[161]
miR-141	Diagnosis	[154]
miR-143	Diagnosis, prognosis (↓)	[43, 51, 156]
miR-145	Diagnosis, prognosis (↓)	[43, 49–51, 154, 162]
miR-146a/b-5p	Diagnosis, prognosis (↓)	[163]
miR-183-96-182 cluster	Diagnosis, prognosis	[56, 59, 66, 154, 164]
miR-187	Diagnosis	[156]
miR-195	Diagnosis (↓)	[21]
miR-200a	Treatment outcome	[56]
miR-203	Diagnosis, prognosis (↓)	[164]
miR-214	Diagnosis	[154]
miR-221	Diagnosis, prognosis (↓), treatment outcome	[55, 154]
miR-222	Diagnosis	[154]
miR-342-3p	Diagnosis (↑)	[21]
miR-375	Diagnosis (↑)	[43, 154]
miR-519d	Prognosis (↑), treatment outcome	[67]
miR-616	Diagnosis (↑)	[165]
miR-622	Diagnosis (↑)	[21]
miR-647	Prognosis (↓), treatment outcome	[67]
miR-720	Diagnosis	[154]
miR-768-3p	Diagnosis	[154]
miR-1256	Diagnosis (↓)	[158]

Arrows indicate the sense of deregulation: (↑): upregulation; (↓): downregulation in PCa versus normal tissues or low risk versus high risk PCa.

**Table 2:** Proteins as PCa biomarkers in FFPE tissue.

Protein	Description	Clinical significance	References
PSA	Prostate-specific antigen	Diagnosis (prostatic metastasis)	[84]
P501S	Prostein	Diagnosis (prostatic metastasis)	[84, 85]
PSCA	Prostate stem cell antigen	Diagnosis (incl. metastasis), prognosis (↑)	[86–88]
AMACR/P504S	*α*-methylacyl-CoA racemase	Diagnosis (↑)	[89–91, 94, 110, 114]
HMWCK	High-molecular-weight cytokeratin	Diagnosis (↓)	[101, 103–105, 110]
ANXA3	Annexin A3	Prognosis (↓)	[166]
CgA	Chromogranin A	Prognosis (↑), treatment outcome	[144–147]
OPN	Osteopontin	Prognosis (↑)	[167]
ZAG	Zinc-alpha 2-glycoprotein	Prognosis (↓)	[168, 169]
PSMA	Prostate-specific membrane antigen	Diagnosis, prognosis (↑)	[170, 171]
GOLPH2	Golgi phosphoprotein 2	Diagnosis (↑)	[99, 100]
GST-pi	Glutathione-S-transferase-pi	Diagnosis (↓)	[172]
HPN	Hepsin	Diagnosis (↑)	[173, 174]
Maspin	Maspin protein/Serpin B5	Diagnosis (↓, aberrant nuclear distribution)	[172, 175, 176]
MMP9	Matrix metallopeptidase 9	Prognosis (↑)	[177, 178]
PDEF/hPSE	Prostate-derived Ets transcription factor	Diagnosis, prognosis (↓)	[177, 179, 180]
SPINK1/TATI	Serine protease inhibitor Kazal-type 1/Tumor-associated trypsin inhibitor	Diagnosis, prognosis (↑)	[181, 182]
Ki67	Antigen KI-67	Prognosis (↑), treatment outcome	[121, 122, 183]
B7-H3 and B7x	B7 family members	Prognosis (↑)	[131, 133]
p53	Cellular tumor antigen p53	Diagnosis (↑), treatment outcome	[134, 135]
p27	Protein p27	Prognosis (↓)	[184, 185]
p16	Protein p16	Prognosis (↓), treatment outcome	[186, 187]
uPA	Urokinase-type plasminogen activator	Prognosis (↑), treatment outcome	[188, 189]
MCT2	Monocarboxylate transporter 2	Diagnosis (↑)	[190]
AR	Androgen receptor	Prognosis (↓), treatment outcome	[119, 191]
PTEN	Phosphatase and tensin homologue	Prognosis (↓), treatment outcome	[115, 117–119, 183]
MSMB/PSP94	*β*-Microseminoprotein/prostate secretory protein 94	Prognosis (↑)	[192]
EZH2	Histone-lysine N-methyltransferase	Prognosis (↑)	[185, 193]
HSP27	Heat shock 27 kDa protein	Prognosis (↑)	[194, 195]
ErbB2/HER2	Receptor tyrosine-protein kinase erbB-2	Prognosis (↑)	[196, 197]
NKX3.1	Homeobox protein Nkx-3.1	Diagnosis (prostatic metastasis)	[198]
c-Myc	Myc protooncogene protein	Prognosis (↑), treatment outcome	[183, 185]
BIRC5	Baculoviral IAP repeat-containing protein 5/Survivin	Prognosis (↓)	[199, 200]
KLK4	Kallikrein-4	Prognosis (↓)	[201]
TERT	Telomerase reverse transcriptase	Prognosis (↑), treatment outcome	[202, 203]
CRISP3	Cysteine-rich secretory protein 3	Diagnosis, prognosis (↑), treatment outcome	[95, 204]
BCL2	Apoptosis regulator Bcl-2	Prognosis (↑), treatment outcome	[139–141]
COX2	Prostaglandin G/H synthase 2	Treatment outcome	[124, 128, 130]
VEGF-A	Vascular endothelial growth factor A	Prognosis (↑), treatment outcome	[205, 206]
HIF-1*α*	Hypoxia-inducible factor 1-alpha	Treatment outcome	[207, 208]

Arrows indicate the sense of deregulation: (↑): upregulation; (↓): downregulation in PCa versus normal tissues or low risk versus high risk PCa.
